# Local critical stress correlates better than global maximum stress with plaque morphological features linked to atherosclerotic plaque vulnerability: an *in vivo *multi-patient study

**DOI:** 10.1186/1475-925X-8-15

**Published:** 2009-08-03

**Authors:** Dalin Tang, Zhongzhao Teng, Gador Canton, Thomas S Hatsukami, Li Dong, Xueying Huang, Chun Yuan

**Affiliations:** 1 Worcester Polytechnic Institute, Mathematical Sciences Department 100 Institute Road, Worcester, MA, USA; 2 University of Washington, Department of Radiology, Seattle, WA 98195 USA; 3 University of Washington, Division of Vascular Surgery, Seattle, WA 98195 USA

## Abstract

**Background:**

It is believed that mechanical stresses play an important role in atherosclerotic plaque rupture process and may be used for better plaque vulnerability assessment and rupture risk predictions. Image-based plaque models have been introduced in recent years to perform mechanical stress analysis and identify critical stress indicators which may be linked to rupture risk. However, large-scale studies based on *in vivo *patient data combining mechanical stress analysis, plaque morphology and composition for carotid plaque vulnerability assessment are lacking in the current literature.

**Methods:**

206 slices of *in vivo *magnetic resonance image (MRI) of carotid atherosclerotic plaques from 20 patients (age: 49–71, mean: 67.4; all male) were acquired for model construction. Modified Mooney-Rivlin models were used for vessel wall and all plaque components with parameter values chosen to match available data. A morphological plaque severity index (MPSI) was introduced based on *in vivo *plaque morphological characteristics known to correlate with plaque vulnerability. Critical stress, defined as the maximum of maximum- principal-stress (Stress-P_1_) values from all possible vulnerable sites, was determined for each slice for analysis. A computational plaque stress index (CPSI, with 5 grades 0–4, 4 being most vulnerable) was defined for each slice using its critical stress value and stress interval for each CPSI grade was optimized to reach best agreement with MPSI. Correlations between CPSI and MPSI, plaque cap thickness, and lipid core size were analyzed.

**Results:**

Critical stress values correlated positively with lipid core size (r = 0.3879) and negatively with cap thickness (r = -0.3953). CPSI classifications had 71.4% agreement with MPSI classifications. The Pearson correlation coefficient between CPSI and MPSI was 0.849 (p < 0.0001). Using global maximum Stress-P_1 _value for each slice to define a global maximum stress-based CPSI (G-CPSI), the agreement rate with MPSI was only 34.0%. The Pearson correlation coefficient between G-CPSI and MPSI was 0.209.

**Conclusion:**

Results from this *in vivo *study demonstrated that localized critical stress values had much better correlation with plaque morphological features known to be linked to plaque rupture risk, compared to global maximum stress conditions. Critical stress indicators have the potential to improve image-based screening and plaque vulnerability assessment schemes.

## Introduction

Atherosclerotic plaques may rupture without warning and cause acute cardiovascular syndromes such as heart attack and stroke. Currently, screening and diagnosis of patients with atherosclerotic plaques are based on medical images such as magnetic resonance image (MRI), ultrasound, intravascular ultrasound (IVUS), or computerized tomography (CT). Increasing evidences showed that such image-only screening and diagnostic techniques are insufficient to identify those victims before the event occurs [1,2]. It has been hypothesized that mechanical forces (rupture triggers) play an important role in plaque rupture process and should be considered in an integrated way with plaque morphology and composition for possible improvement of plaque assessment schemes [3]. Large-scale studies based on *in vivo *patient data combining mechanical stress analysis, plaque morphology and compositions are needed to identify the critical stress indicators that are linked to plaque vulnerability.

In recent years, MRI techniques have shown their ability to non-invasively determine plaque size, shape and component [4-6]. Yuan et al. developed multi-contrast techniques to improve the quality of MR-images and to better differentiate various components of the plaque [4,6]. Attempts of using ultrasound and IVUS techniques have been made to quantify vessel motion, mechanical properties and vessel wall structure, even to predict rupture locations [7,8]. Using non-invasive MRI techniques, Cai et al. developed a classification system for carotid plaques based on *in vivo *MRI [9]. MRI- and histology-based computational simulations for plaque rupture investigation and vulnerability assessment have been conducted by several groups and many interesting and significant results have been reported [10-24]. Holzapfel et al. introduced multi-layer 3D models for the simulation of balloon angioplasty using MRI and direct mechanical testing [10]. Steinman studied influence of complex vessel geometry on flow behaviors using image-based realistic arterial geometries [11]. Weinbaum et al. investigated critical stress behaviors at plaque cap with micro-calcification inclusions and elevated stress levels were observed [16]. Bluestein et al. introduced fluid-structure interaction models to study influence of microcalcifications on vulnerable plaque mechanics [17]. Li et al. showed that wall stress calculated based on *in vivo *MRI of carotid arteries was higher in symptomatic patients than in asymptomatic patients [19]. Groen and Wentzel et al. reported a follow-up case study showing high flow shear stress region was associated with site of plaque rupture [21]. Tang et al. introduced a "local maximum stress hypothesis" to identify the critical site and stress conditions in the plaque and proposed an *ex vivo *MRI-based computation plaque vulnerability index (CPVI) to access plaque vulnerability [12]. Results from 34 2D *ex vivo *MRI slices from 14 human atherosclerotic coronary arteries indicated that CPVI has a good agreement (89%) with histology-based assessment.

Most of the current computational studies were at model development stage using one or a few samples. Large-scale *in vivo *MRI-based plaque mechanical analysis is lacking in the current literature. Due to the complexity of plaque architecture, analyzing the large amount of computational stress/strain data and identifying critical stress indicators which correlate closely with plaque vulnerability are time-consuming and challenging. A validated easy-to-use stress index would be more practical for potential patient screening and clinical applications.

In this paper, we extend our previous ex vivo study to an *in vivo *MRI-based multi-patient study to further validate that it is the localized critical stress conditions, not global maximum stress conditions, that have better correlations with plaque morphological features known to be linked to plaque vulnerability. A morphological plaque severity index (MPSI) and a computational plaque stress index (CPSI) were introduced for plaque classification and comparison. Correlations between CPSI and MPSI, plaque cap thickness, and lipid core size were quantified.

## MRI data, method and model

### MRI Data Acquisition

**Figure 1:**
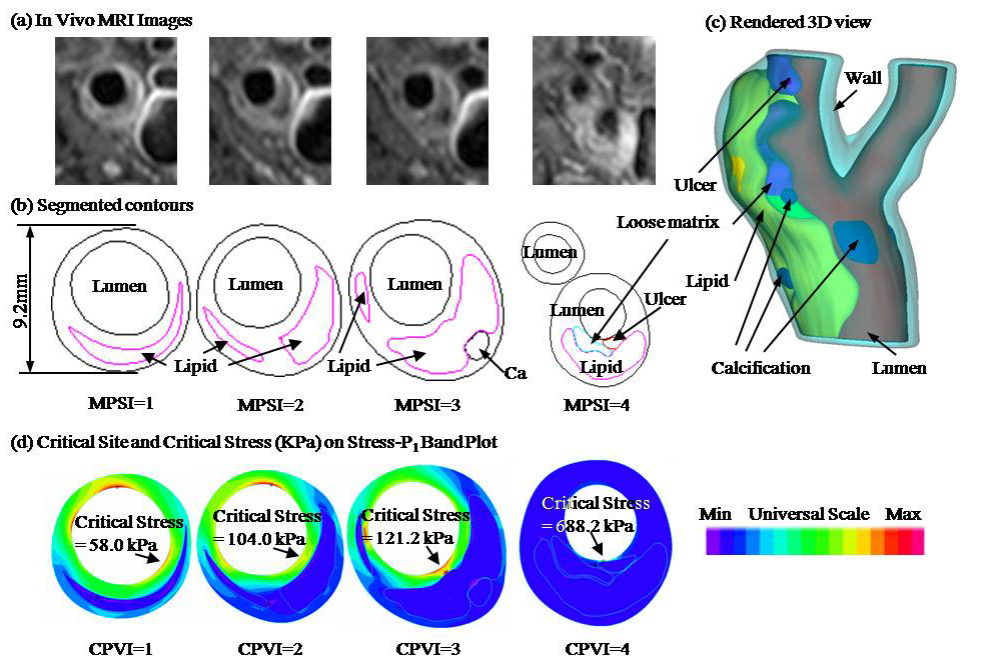
**(a) *In vivo *MR-images (Lumen is marked by a red asterisk)**; (b) Segmented contour plots showing plaque components (Black: Lumen, vessel wall or calcification; Magenta: Lipid core; Red: Ulcer); (c): Rendered 3D view using the segmented contours; (d): Band plot of maximal principal stress (Stress-P_1_) of corresponding slices; CPSI values were calculated based on critical Stress-P_1 _values at critical sites, following procedures described in the paper.

Two hundred and six (206) high resolution *in vivo *MRI slices of carotid atherosclerotic plaques from 20 patients (age: 49–71, mean age: 67.4; all male) were provided by Vascular Imaging Laboratory of the University of Washington (UW) using protocols approved by UW Institutional Review Board and segmented by a self-developed software package (CASCADE) [25]. Due to limited resources and difficulty in getting healthy volunteers, each 2D slice was treated as an independent case to gain a good representation of all lesion types. Patients were imaged with a 1.5-T MR scanner (Signa Horizon EchoSpeed, General Electric Health Care). Precontrast MR images that included double-inversion-recovery T1W, proton density-weighted (PDW), T2W, TOF, and postcontrast double-inversion-recovery T1W MR images of carotid arteries were obtained with a previously published standardized protocol (T1W: repetition time/echo time/inversion time, 800/10/650 ms; PDW and T2W: repetition time/echo time, 3RR, 20/40 ms; TOF: repetition time/echo time, 23/3.8 ms) [4-6]. Plane and space resolution was at 0.31 × 0.31 × 2.0 mm^3 ^(with scanner interpolation). Figure 1 gives 4 selected MRI slices with segmented contour plots and corresponding 3D rendered geometry of the plaque.

### Assignment of Morphological Plaque Severity Index (MPSI)

Since histological data is in general not available for *in vivo *studies, a morphological plaque severity index (MPSI) was introduced (Table 1) and assigned to each segmented MRI slice based on plaque morphological features known to correlate with plaque vulnerability from histopathological studies [3,9,12]. These features include: 1) the size and distribution of the soft lipid rich necrotic core (LRNC); 2) the fibrous cap thickness (which correlates with plaque stability); and 3) the presence of ulcer, intraplaque hemorrhage and thrombi. MPSI values (0, 1, 2, 3 to 4) indicate the level of increasing severity. The MPSI definitions are closely associated with the AHA (American Heart Association) lesion type classifications (see Table 1) and the representative morphologies are shown in Figure 1(b).

**Table 1:** Morphological plaque vulnerability index (MPSI) classifications and comparison with AHA classifications

**MPSI Category**	**Corresponding AHA lesion types (modified)**	**Description**	**Level of vulnerability**
0	I or II	Normal or nearly normal wall.	Very stable

1	III	Moderate intimal thickening, no extracellular lipid, calcification or significant inflammation.	Stable

2	IV/V with less than 30% NC by area; or VII; or VIII	Advanced lesion with small necrotic core (<30% of plaque size), or can be fibrotic or calcified, thick fibrous cap (> 200 μm).	Slightly unstable

3	IV/V with 30–40% NC by area	Advanced lesion with Moderate lipid core (30–40% of plaque size) and fibrous cap (150–200 μm).	Moderately unstable

4	IV/V with > 40% NC by area; or VI	Advanced lesion with a very large necrotic core (>40%), thin fibrous cap (<150 μm), or with fibrous cap rupture, ulceration, or intraplaque hemorrhage.	Very unstable

Quantitative plaque lipid core size and cap thickness information will be based on plaque component contour information in this paper. In particular, plaque cap thickness will be calculated as the shortest distance between a lipid core and lumen, while the thin plaque cap may not be directly measurable by MRI.

Introduction of MPSI provides a combined morphological index for plaque classification. This is similar to the histopathological plaque vulnerability index (HPVI) previously proposed in our *ex vivo *study for the vulnerability of coronary plaque [12]. The semi-quantitative MPSI will serve as a "benchmark" to validate the CPSI in this paper. MPSI distributions of the 206 slices are listed in Table 2.

**Table 2:** Case distributions according to MPSI and agreement rate between CPSI and MPSI

**MPSI**	**Number of Slices**	**Percentage (%)**	**Agreement Rate (%)**
0	33	16.02	100

1	45	21.84	64.44

2	42	20.39	57.14

3	40	19.42	57.50

4	46	22.33	82.61

### A Pre-Shrink Process for *In Vivo *Data

**The ***In vivo *imaged arteries were under physiological pressure conditions. Therefore a pre-shrink process was necessary to obtain the zero-pressure geometry, which was used as the numerical starting geometry, and to recover the *in vivo *geometry when pressure was imposed in the lumen. In this study, for each patient, the shrinkage was determined by choosing the most circular (round) slice which would recover its *in vivo *shape best when lumen pressure was imposed. The shrinkage rates of contours of lumen (δ_in_) and outer wall (δ_out_) were numerically determined following an iterative procedure so that: 1) the vessel cross-section area was conserved (conservation of mass); and 2) the pressurized morphology and the original *in vivo *morphology had the best agreement. The determined shrinkage was applied to all the slices of this patient for consistency and uniformity and for future comparison with 3D models. This approach was chosen also because slice-specific shrinkage data would not be available in patient-screening practice. The average inner circumference shrinkage rate (δ_in_) was 12% (SD ± 2.2%) for the 206 2D models.

**Figure 2:**
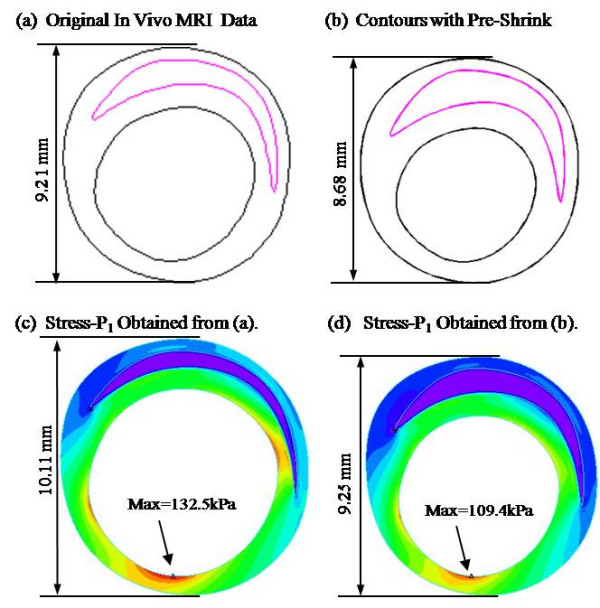
**Geometry and Stress-P_1 _plots of one example showing that the pre-shrink process improves stress predictions**. Patient pressure: Pmean = 121.5 mmHg. Shrinkage: Inner wall: 14%; Outer wall: 5.8%. Maximum Stress-P_1 _over-estimation without pre-shrink: 21%.

Figure 2 gives an example which shows that maximum principal stress (Stress-P_1_) would be over-estimated by 21% if pre-shrink process was not performed. The pressurized vessel dimension with pre-shrink (Figure 2(d)) matched *in vivo *dimension (error < 0.4%). Without pre-shrink, vessel dimension expanded about 10% (Figure 2(c)).

### Mesh Generation, Computational Models and Solution Methods

Model mesh generation, solution and data post-processing were performed using ADINA, a commercial finite element package (ADINA R&D, Inc., Watertown, MA), which has been used by Tang et al. in recent years to solve atherosclerotic artery models [12-15,24]. The computational mesh was made using ADINA automated mesh generation system. Finer meshes were used for thin-cap and large curvature areas to avoid computational artifacts. A typical plaque sample with 4 slices was given by Figure 1, with dimension indicated on one slice. The solid models are given below:

(1)

(2)

(3)

(4)

where **σ **is stress tensor (superscripts indicate different materials), ε is strain tensor, **v **is solid displacement vector, superscript letters "r" and "s" were used to indicate different materials. No-slip conditions and natural traction equilibrium conditions are assumed at all interfaces. For simplicity, all material densities were set to 1 g·cm^-3 ^in this paper.

All plaque components including fibrous tissue, hemorrhage, lipid core, calcification and loose matrix were assumed to be non-linear, isotropic and hyper-elastic. The modified Mooney-Rivlin model was used as the material model [26]:

(5)

(6)

where *I*_1 _and *I*_2 _are the first and second invariants, **C **= [C_ij_] = **X**^T^**X **is the right Cauchy-Green deformation tensor, **X **= [X_ij_] = [∂x_i_/∂a_j_], (x_i_) is the current position, (a_i_) is the original position. Material parameters *c*_*i *_and *D*_*i *_(*i *= 1,2) were chosen to match available experimental measurement data [12,14,23]: vessel material/fibrous cap, *c*_1 _= 36.8 kPa, *D*_1 _= 14.4 kPa, *D*_2 _= 2; lipid core/hemorrhage, *c*_1 _= 2 kPa, *D*_1 _= 2 kPa, *D*_2 _= 1.5; calcification, c_1 _= 368 kPa, D_1 _= 144 kPa, D_2 _= 2.0; loose matrix, *c*_1 _= 18.4 kPa, *D*_1 _= 7.2 kPa; *D*_2 _= 1.5. *c*_2 _= 0 for all materials. A pulsating pressure was imposed in the lumen using the systolic/diastolic arm pressure data for each patient from their last hospital admission. Average systolic and diastolic pressures were 144.7 ± 21.5 and 75.6 ± 13.5 mmHg respectively, for the 20 patients studied. Pressure at the out-boundary of each vessel slice was set to zero.

The computational plaque models were solved using ADINA which uses unstructured finite element methods, nonlinear incremental iterative procedures and the Newton-Raphson iteration method to deal with complex geometries and nonlinear systems. Details of the models and methods are given in Tang et al. [13] and Bathe [26].

### Definition and Calculation of Critical Stress

Critical stress was defined as the maximum of all local maximum Stress-P_1 _values from possible vulnerable sites. It is known that thin plaque cap is closely related to plaque rupture. Thus vulnerable sites include all locations where a thin region covers a plaque component and a local maximum Stress-P_1 _was found. It should be noted that our "thin region" includes fibrous cap over a lipid core, as well as "cap" over calcification and other plaque components. Healthy sites where no plaque components were present and rupture was unlikely were excluded from the "vulnerable site list", even if a local stress maximum occurred there. For each slice, Stress-P_1 _distribution corresponding to peak pressure condition was obtained from the 2D computational model. An automatic search was performed to find all local maximum Stress-P_1 _values from vulnerable sites. Then critical stress for that slice was determined using the definition given above. The site where critical stress was found was defined as the critical site. For slices without any components, critical stress was set to zero since these slices are assumed to be very stable.

### CPSI Assignment, Quantitative Definition of Morphological Features and Data Analysis

Each slice was assigned a CPSI value (0, 1, 2, 3 or 4) according to its critical stress by using five stress intervals, which were determined to have best match rate with MPSI. Cap thickness was defined as the shortest distance between the critical site and the nearest plaque component contour. Normalized lipid index (NLI) and normalized wall index (NWI, also called plaque burden) are defined as:

(7)

(8)

Correlations between CPSI values and MPSI, plaque cap thickness, normalized lipid index and normalized wall index were analyzed. Global maximum Stress-P_1_values for each slice were also recorded for comparison and correlation analyses.

## Results

### Correlations between Critical Stress and Cap Thickness, Normalized Lipid Index and Plaque Burden

**Figure 3:**
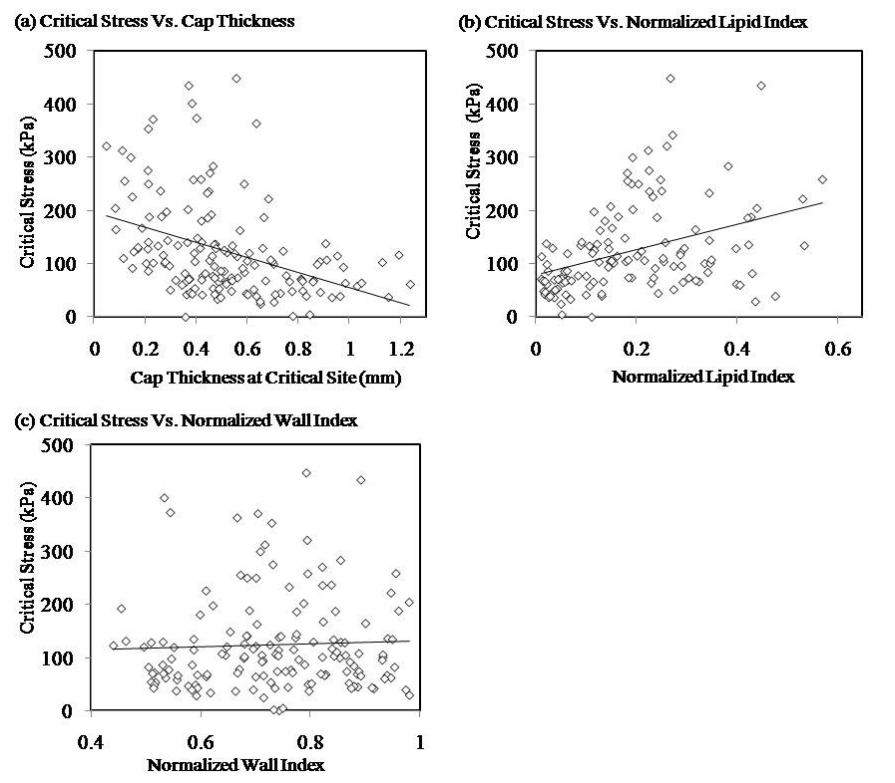
**Critical stress values correlate much better with MPSI than global maximum values of Stress-P_1 _from 206 carotid 2D plaque samples (*in vivo *MRI)**.

Excluding slices without any plaque components and slices with calcification component only, we have 152 slices for this analysis. All morphological quantities were calculated based on segmented MRI data with interpolations as needed in the modeling process. Figure 3 gives plots of critical stresses vs. plaque cap thickness, NLI, and NWI, respectively. Critical stress values correlated with normalized lipid index positively (r = 0.3879) and correlated with cap thickness negatively (r = -0.3953). No correlation was found between critical stress values and NWI (Pearson correlation coefficient r = 0.0444).

### Correlation between CPSI and MPSI

A simple numerical code was used to determine five equal stress intervals [0, a), [a, 2a), [2a, 3a), [3a, 4a), and [4a, +8) corresponding to CPSI values 0–4 to reach the best agreement between CPSI and MPSI. The five intervals (unit: KPa) [0, 35.5), [35.5, 71), [71, 106.5), [106.5, 142), and [142, +8) were used for CPSI values of 0, 1, 2, 3 and 4, respectively. The optimized agreement rate was 71.4% (147 matching cases out of 206). The Pearson correlation coefficient between CPSI and MPSI was 0.849 (p < 0.0001). Table 2 lists number of cases and agreement rate for each MPSI grade group. The agreement rate was 82.6% for the highly vulnerable group (MPSI = 4). According to the CPSI stress intervals, a plaque will be considered highly vulnerable (high risk) if its critical stress is higher than 142 kPa.

### Correlation between an Index Based on Global Maximum Stress-P_1 _Values and MPSI

**Figure 4:**
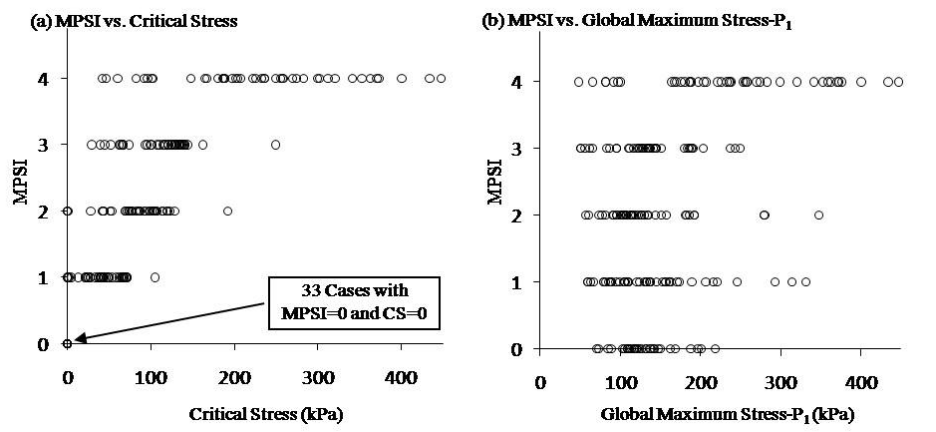
**Critical stress values correlated with cap thickness at critical site negatively (Pearson correlation coefficient r = 0.3953) and normalized lipid index positively(r = 0.3879)**. No correlation between critical stresses and the normalized wall index was found (r = 0.0444).

Using global maximum Stress-P_1 _value for each slice to calculate a global maximum stress-based CPSI (G-CPSI), the agreement rate between G-CPSI and MPSI was 34.0%. And the Pearson correlation coefficient was 0.209. Figure 4 shows that critical stress values correlate much better with MPSI compared to global maximum Stress-P_1 _values. This is not surprising because global maximal stress often appears at healthy parts of the vessel where either the vessel wall is thinner than the diseased plaque side or the vessel curvature is large, and is not a good indicator for plaque vulnerability assessment [12].

## Discussion

### Purpose of Introducing Localized Critical Stress Indicator and CPSI

The purpose of introducing the localized critical stress indicator and CPSI index is to identify stress indicators which are more closely linked to plaque vulnerability, compared to global maximum stress conditions. The critical-stress based CPSI provides a plaque classification and assessment scheme which includes mechanical factors, plaque morphological features and tissue compositions for possible patient-screening applications. A 5-point scale (CPSI = 0, 1, 2, 3, and 4) was used so that the CPSI-based classifications would be comparable with AHA plaque classification scheme (Table 1). One main potential advantage for introducing CPSI is that it can provide quantitative assessment of plaques, compared to the current qualitative AHA scheme. More refined scales using decimals may be introduced to provide more quantitative assessment. Results from 206 slices suggested that CPSI and MPSI had a good agreement on plaque classification. Global maximum stress values showed much weaker correlation with MPSI, suggesting that localized critical stress may be a better indicator for plaque vulnerability.

The disagreement (28.6% overall, 17.4% for high risk plaques) suggested that CPSI scheme may complement image-only assessment schemes and lead to potential improvements. The present study is the first large-scale (n = 206) case study quantifying differences between mechanics-image combined and morphology-only assessment schemes. In Table 2, the worst match between CPSI and MPSI is in the scale of 1–3. This range of plaque represents the moderate stenosis, but with high risk for future plaque rupture. In fact, this class of plaque is the most interesting plaque that is often ignored in clinical practices. Developing some biomarker to identify the risk of these plaques would be great benefit for clinicians. CPSI could serve as such a biomarker providing additional mechanical stress information for image-based plaque assessment schemes.

It should be understood that plaque rupture is a multi-faceted process. CPSI covers only mechanical and morphological factors. We hope CPSI could provide complementing information for plaque assessment that image alone could not provide. Multiple biomarkers from different channels such as cell activity, lumen surface condition and inflammation should be jointly considered for more complete and accurate vulnerability assessment.

### 2D vs. 3D Models and Model limitation

**Figure 5:**
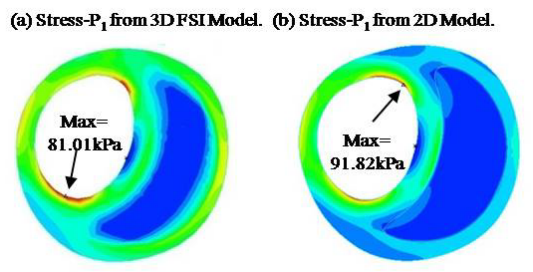
**Stress-P_1 _distributions from 2D and 3D FSI models have similar patterns with 2D model giving higher maximum Stress-P_1 _on the lumen**.

We used 2D models in the evaluation of CPSI for commercialization and clinical application purposes. With current modeling and computing power, only 2D modeling and mechanical analysis are practical. It takes only a few minutes with our automated procedure to make one 2D model, while it takes several weeks for an experienced researcher to construct a 3D fluid-structure interaction (FSI) model which is impractical for clinical applications. 2D and 3D models were compared in our previous studies and it was found that 2D models could provide good approximations to 3D models in correlation and classification studies [13]. While 2D and 3D models do give different Stress-P_1 _values, Figure 5 shows that they have similar distribution patterns and the majority of those values differ in a proportional way. Stress values from 2D and 3D plaque models can differ for the following reasons: a) 3D model includes longitudinal stretch which leads to overall (3D mean) increased 3D Stress-P_1 _values; b) At the same time, 2D model expands more in radial direction when pressurized because it has no axial stretch and no bonding effect from neighboring slices. That leads to higher Stress-P_1 _value at inner wall (lumen) and lower Stress-P_1 _values at outer portion of the wall as shown by Figure 5c;) 3D longitudinal curvature of the vessel can lead to greater 2D/3D stress prediction differences.

Several other limitations exist in this study: a) Patient-specific material properties were not included because data was not available with current technology; b) some important factors such as lumen surface inflammation and erosion conditions were not taken into account since current *in vivo *MRI technology could not accurately provide these data; c) residual stress (opening angle) was not included in our model; d) the CPSI cannot well estimate the risk of cases with very narrow lumen and thick fibrous cap but having large lipid core and other components. Accuracy of CPSI could be improved with accurate cap thickness and material property measurements.

## Conclusion

Results from this multi-patient *in vivo *study based on 206 slices demonstrated that localized critical stress values had much better correlation with plaque morphological features known to be linked to plaque rupture risk (r = 0.849 with MPSI), compared to global maximum stress conditions (r = 0.209 with MPSI). Critical stress values correlated positively with normalized lipid index (r = 0.3879) and negatively with cap thickness (r = -0.3953). Large scale and long-term patient studies using 3D models are needed to further validate our findings and identify potential stress risk indicators for patient screening plaque vulnerability assessment.

## Competing interests

Other than the grants listed in the acknowledgement section, the authors declare that they have no other competing interest.

## Authors' contributions

DT, ZT, and XH were responsible for computational modeling and data analysis part. CY, GC, TSH, and LD were responsible for MRI data acquisition and segmentation part. All authors 1) have made substantial contributions to conception and design, or acquisition of data, or analysis and interpretation of data; 2) have been involved in drafting the manuscript or revising it critically for important intellectual content; and 3) have given final approval of the version to be published. Each author has participated sufficiently in the work to take public responsibility for appropriate portions of the content.

## Authors' information

Tang's group has been publishing image-based modeling work in recent years. For more information, please visit Tang's website: . Dr. Yuan's group and their lab (Vascular Imaging Laboratory, University of Washington) have been developing MR imaging methods and have published extensively in this area. For more information, please visit their website: .
